# Mixed Gonadal Dysgenesis: A Comprehensive Review of Clinical Spectrum, Diagnostic Strategies, and Management Approaches

**DOI:** 10.1111/cen.70053

**Published:** 2025-11-09

**Authors:** Dinesh Giri, Sushil Yewale, Hannah Hickingbotham, Cara Williams, Mohamed Shalaby, Julie Alderson, Julie Park

**Affiliations:** ^1^ Bristol Royal Hospital for Children Bristol UK; ^2^ University of Bristol Bristol UK; ^3^ Alder Hey Children's Hospital Liverpool UK; ^4^ Croydon University Hospital London UK; ^5^ University of Liverpool Liverpool UK

**Keywords:** differences in sex development, gonadal tumour, mixed gonadal dysgenesis

## Abstract

**Background:**

Mixed gonadal dysgenesis (MGD) is a rare form of differences in sex development (DSD) typically associated with 45,X/46,XY mosaicism. The phenotypic presentation of MGD varies from atypical genitalia to typical male or female appearances often associated with Turner stigmata. Some of the challenges in the clinical management of patients with MGD include gonadal malignancy risk, decisions on gonadectomy, fertility and sex of rearing. The management is predominantly multidisciplinary with a focus on patient and family centred care.

**Methods:**

This article was prepared as a narrative review based on a comprehensive search of the literature. A systematic search of the PubMed, Embase, Scopus, and Google Scholar databases was performed using the key terms *“mixed gonadal dysgenesis,” “45,X/46,XY mosaicism,” “differences in sex development,” and “gonadal tumour risk”* to identify relevant articles published between 2000 and 2024. References from the identified papers were further screened to capture additional relevant literature. We gathered the findings to provide an updated overview of MGD, focusing on epidemiology, clinical manifestations, diagnostic evaluation, malignancy risk, approaches to management, psychosocial considerations, and evolving strategies in the long‐term care of patients with MGD.

**Results:**

MGD accounts for 5%–15% of cases of atypical genitalia and carries a 15%–25% risk of gonadal tumour, with the highest malignancy rates in intra‐abdominal gonads. Approximately 12%–15% of patients with MGD may experience gender incongruence later in life. Management has shifted from early surgical intervention to a multidisciplinary, patient‐centred, and shared decision making approach.

**Conclusions:**

The future care of patients with MGD is likely to include biomarker‐driven surveillance, along with advanced fertility preservation techniques. Long‐term outcome data for patients with MGD along with patient‐reported outcomes, are limited in the literature, underscoring the need for further research.

## 1 Introduction

Mixed gonadal dysgenesis (MGD), first described in 1963 [1], represents a rare form of differences in sex development (DSD) resulting from 45,X/46,XY mosaicism and is typically characterised by the presence of a dysgenetic testis at various levels of descent on one side and a streak gonad on the other [2]. The most common karyotype reported is 45,X/46,XY mosaicism. Other rare karyotypes described include 45,X/47,XXY, and 45,X/47,XYY [3]. Rarely, cases with clinical features resembling MGD have been reported despite a normal 46, XX or 46, XY karyotype, often attributed to low‐level mosaicism detectable only with advanced genetic techniques [4].

The phenotypic features of patients with MGD are heterogeneous and range from atypical genitalia to predominantly male or female characteristics with Turner stigmata [5, 6]. Many individuals have Müllerian remnants on the same side as the streak gonad, whereas the most common phenotypic finding is proximal hypospadias with perineal division of the corpus spongiosum and ventral curvature [7].

MGD is one of the most frequent causes of DSD after congenital adrenal hyperplasia (CAH) [8]. Historically, assignment of female sex of rearing following early prophylactic gonadectomy to reduce the risk of gonadoblastoma was common practice [9]. However, more recent clinical approaches focus on patient‐centred care and shared decision‐making processes between the patient, family, and health care professionals while delaying irreversible surgical interventions until a comprehensive multidisciplinary team (MDT) plan has been formulated with the family [10, 11]. The European Society for Paediatric Endocrinology (ESPE), along with other recent guidelines, emphasise that management decisions should be personalised to balance the need for malignancy risk reduction with the preservation of potential future fertility and psychological well‐being [11, 12]. This review outlines the epidemiology, clinical features, diagnostic evaluation, treatment options and psychosocial aspects of paediatric MGD while integrating current evidence and recommended guidelines.

## 2 Epidemiology

MGD is rare, with an estimated incidence of 1 in 15,000 to 1 in 30,000 live births [13]. The prevalence of MGD may be underestimated due to its wide range of phenotypic presentations. Among infants evaluated for atypical genitalia, MGD accounts for approximately 5%–15% of cases [14]. The condition emerges from early embryonic errors in chromosomal arrangements resulting in a mixture of 45,X/46,XY cell lines [15]. The phenotype of patients with MGD is variable and determined by the extent and degree of mosaicism across different tissues, including the gonads and genital structures [16]. Prenatal diagnostic tests such as noninvasive prenatal testing (NIPT) and amniocentesis can detect 45,X/46,XY mosaicism in foetal cells, but these findings do not reliably predict the postnatal phenotype of the infant [17].

## 3 Clinical Presentations

The clinical spectrum of MGD to an extent is influenced by the functional capacity of the dysgenetic gonad along with the degree of androgen exposure during critical periods of foetal development [18]. The clinical presentations are summarised in Table 1.

**Table 1:** Clinical presentations of MGD.

Neonatal period	Childhood or adolescence period	Turner stigmata
Atypical genitalia:	Short stature Primary amenorrhea Absent secondary sexual characteristics Infertility*	Short stature Webbed neck Shield chest Cubitus valgus High arched palate Renal malformations (horseshoe kidney) Cardiac anomalies (bicuspid aortic valve, coarctation of aorta)
Clitoro‐phallus of moderate size		
Severe hypospadias		
Bifid scrotum		
Undescended or partially descended gonads		
Varying degrees of labioscrotal fusion		

* Usually presents in adulthood.

### 3.1 Neonatal Presentation

In the neonatal period, the most common presentation is with atypical genitalia. The assessment of external genitalia reveals broad phenotypic patterns that include a phallus of moderate size, severe hypospadias with a perineal or scrotal meatus, significant ventral curvature of the phallus, penoscrotal transposition, and undescended or partially descended gonads with varying degrees of labioscrotal fusion giving the appearance of a bifid scrotum [19]. In most infants, a gonad is usually palpable within one labioscrotal fold, and on the contralateral side, there is a non‐palpable streak gonad [19]. The gonadal locations in these patients can vary with testes often located in the scrotum or within the inguinal canal while streak gonads typically remain intra‐abdominal. The gonad on one side is typically a streak gonad, while the contralateral gonad is usually a dysgenetic testis. Occasionally, the contralateral gonad may appear morphologically normal and descend into the scrotum, but histological studies have shown that even apparently normal testes can harbour varying degrees of dysgenesis and carry a persistent, although lower, tumour risk [13, 19]. The secretion of Anti‐Müllerian Hormone (AMH) is often impaired by the dysgenetic testis thus leading to persistence of some Müllerian structures [13]. The most common observed remnants are a hemi‐uterus with associated ipsilateral fallopian tube or prostatic utricle [7]. The affected infants may also have either a blind‐ending vaginal pouch or a urogenital sinus reflecting incomplete differentiation of urogenital tract [7]. The phenotype of MGD ranges from minimal virilisation in females such as clitoral enlargement, to extreme undervirilisation in males, with severe hypospadias leading to atypical genitalia [18].

### 3.2 Turner Stigmata

Some patients with MGD and 45,X/46,XY mosaicism may exhibit phenotypic characteristics that are similar to Turner syndrome. Short stature is common in individuals regardless of their phenotypic sex. The other features include webbed neck, shield chest, cubitus valgus, high‐arched palate, renal malformations such as horseshoe kidney, and cardiac anomalies such as bicuspid aortic valve and coarctation of aorta. Given these associations, it is recommended that a comprehensive evaluation including renal ultrasonography, together with a complete cardiac evaluation with echocardiographic assessment should be performed in all children with 45,X/46,XY karyotype to facilitate early detection and management of these anomalies [20].

### 3.3 Typical Male and Female Presentations

Although most individuals with MGD present with atypical genitalia, some individuals 45,X/46,XY genotype may have more typically developed genitalia. A male newborn may present with hypospadias or one undescended testis as the only genital abnormality, while some 45,X/46,XY individuals have streak gonads and no virilisation, resembling females with 45,X Turner syndrome. These patients often remain unidentified until later in childhood or adolescence, when they present with short stature, primary amenorrhea or absent secondary sexual characteristics [21]. Some individuals with mosaicism may have functional ovarian tissue and have been known to undergo spontaneous pubertal development with menstruation, although this occurs rarely [21]. Infertility can be the only presenting problem which is often diagnosed in adulthood.

## 4 Diagnostic Evaluation

The diagnosis of DSD in infants requires examination of their external genitalia supported by detailed endocrine and genetic investigations. A summary of investigations is shown in Table 2. A high index of suspicion is needed in individuals with Turner‐like features and virilisation.

**Table 2:** Clinical investigations for assessment of patients with potential MGD.

Investigation	How it aids diagnosis
*Chromosomal Analysis*	
QfPCR	Detects presence of SRY gene
Chromosomal microarray	Detects tissue mosacisim and any structural Y chromosome defects
Targeted karyotype or FISH analysis of gonadal tissue or buccal/skin fibroblasts	Pursued if ongoing clinical suspicion with normal karyotype
*Imaging studies*	
Ultrasound pelvis	Identification of internal reproductive structures
MRI pelvis	May be required if ultrasound not sufficient
Ultrasound abdomen	To assess for associated renal anomalies
Echocardiogram	To assess for associated cardiac anomalies
*Endocrine evaluation*	
Serum electrolytes	To exclude CAH, salt‐wasting crisis and adrenal insufficiency
Cortisol	
17‐hydroxyprogesterone (17‐OHP)	
Testosterone	May be low‐normal in infants with MGD
hCG stimulation test	To assess the ability of the testicular tissue to mount a testosterone response if baseline testosterone is low or outside mini‐puberty window
Anti‐Müllerian hormone (AMH)	Usually low‐normal or in female range in MGD
LH	May be high due to hypergonadotropic state from non‐functional dysgenetic gonads
FSH	
Examination under anaesthesia(EUA): cystoscopy/vaginoscopy	Useful to accurately visualize the external and internal genital anatomy
Laparoscopy	Enables definitive anatomical delineation and also allows for targeted gonadal biopsy or removal

Newborns with atypical genitalia need prompt and comprehensive diagnostic assessment. The initial key steps in the evaluation include:

### 4.1 Chromosomal and Genetic Testing

Quantitative fluorescent polymerase chain reaction (QfPCR) is often performed as a first‐line investigation to detect the presence of the *SRY* gene (sex‐determining region Y). Chromosomal analysis (standard karyotype) via peripheral blood remains the cornerstone for diagnosing MGD. A result showing a mosaic 45,X/46,XY karyotype confirms the diagnosis in most cases. Standard cytogenetic techniques can quantify the relative proportions of 45,X vs 46,XY cell lines in the peripheral blood lymphocytes. Karyotyping can also identify numerical and structural abnormalities such as 45,X/46,XY mosaicism, isodicentric Y chromosome, and translocations. Chromosomal microarray (comparative genomic hybridisation [CGH] array or SNP array) provides higher sensitivity for detecting low‐level mosaicism and submicroscopic copy number variations (CNVs) but cannot identify balanced structural rearrangements. Since mosaicism can vary between tissues, the karyotype in the blood might not accurately reflect the gonadal karyotype. Targeted karyotype or Fluorescent *in situ* hybridisation (FISH) analysis of buccal epithelia cells obtained via swab, skin fibroblasts or gonadal tissue (if having gonadectomy for clinical reasons) may be required when clinical suspicion persists after normal karyotype results.

When a 45,X/46,XY karyotype is identified prenatally by amniocentesis or chorionic villus sampling testing, postnatal confirmation by cytogenetic testing is essential to guide clinical management [21].

### 4.2 Imaging Studies

The identification of internal reproductive structures is a critical part of diagnostic evaluation and is often achieved by pelvic ultrasound performed during the first week of life. In individuals with MGD, ultrasound may show the presence of a uterus or uterine horn, which may be hypoplastic. It can also localise gonads, which might include a streak gonad appearing as an undifferentiated tissue in the pelvic area and a dysgenetic testis located in the inguinal canal or abdomen. Ultrasound of the abdomen is also useful to assess the kidneys, given the possible association with renal anomalies [22]. Pelvic ultrasound is typically the first‐line for localisation of gonads and identification of Müllerian structures. MRI is reserved for cases where ultrasound findings are inconclusive, as it offers superior soft‐tissue contrast and detailed imaging. However, its accuracy in DSD can be variable [23].

### 4.3 Examination Under Anaesthesia (EUA) and Laparoscopy

EUA, including cystoscopy and vaginoscopy, offers further advantage by accurately visualising the external genital anatomy, urogenital sinus, previously undetected Müllerian structures, or prostatic utricles that are difficult to confirm by imaging alone

While noninvasive imaging methods such as pelvic ultrasound and/or MRI are first‐line tools to assess internal reproductive structures and gonads, they may miss or inadequately characterise intra‐abdominal gonads and Müllerian duct remnants. For example, in children with complex DSD, ultrasound failed to visualise Müllerian duct remnants in approximately 40% of cases, whereas laparoscopy achieved optimal visualization in all patients [23].

Laparoscopy not only enables definitive anatomical delineation but also allows for targeted gonadal biopsy or removal in the same procedure, making it superior to imaging alone for diagnostic accuracy and surgical planning [23].

### 4.4 Endocrine Evaluation

The primary aim of endocrine evaluation is to identify the aetiology of DSD and assessing the function of gonads. The key investigations include: plasma electrolytes, cortisol and 17‐hydroxyprogesterone (17‐OHP) concentrations:

While these are in the normal range in patients with MGD, they are often done to exclude CAH, salt‐wasting crisis and adrenal insufficiency [24].

#### 4.4.1 Assessment of Leydig Cell Function

Phallic size provides a useful first‐line clinical indicator of prenatal androgen exposure and, indirectly, Leydig cell function during fetal development. A phallus of appropriate size for gestational age suggests adequate androgen production in utero. However, phallic size alone cannot fully characterise Leydig cell capacity, particularly outside the mini‐puberty period.

Due to the presence of dysgenetic testis, the plasma testosterone concentrations in MGD patients tend to be low‐normal relative to typical male infants but remain elevated compared with those typically seen in female infants. When measured during the mini‐puberty window, typically in the first 3 months, testosterone elevation can indicate functional leydig cells. If baseline testosterone concentration is low or if being evaluated outside the mini‐puberty window, a human chorionic gonadotropin (hCG) stimulation test can be useful to assess if the testicular tissue can mount an adequate testosterone response. In MGD, due to the presence of dysgenetic testis, this testosterone response to hCG stimulation is often blunted, Müllerian reflecting impaired Leydig cell activity.

#### 4.4.2 Anti‐Müllerian Hormone (AMH)

AMH is produced by Sertoli cells and therefore detectable AMH is a marker for the presence of testicular tissue. AMH concentrations in individuals with MGD are typically toward the lower end of the reference range for healthy male infants with normal testicular function, reflecting relative Sertoli cell dysfunction [25].

#### 4.4.3 Gonadotropins (Luteinising Hormone (LH) and Follicle Stimulating Hormone (FSH))

In neonates, gonadotropins concentrations may be elevated if the dysgenetic gonads are nonfunctional thus resulting in a hypergonadotropic state. However, interpretation in the first few days of life can often be difficult. Therefore, trends over the first few months might show rising FSH concentrations, particularly if there is a substantial 45, X component leading to early gonadal failure [2].

## 5 Management Strategies in MGD

The management of MGD requires a coordinated MDT approach. The primary objective of management is to optimise long‐term physical health, reproductive potential, and psychosocial well‐being through coordinated and integrated care. The core components of care include sex assignment, excisional biopsy of the dysgenetic or streak gonads to decrease tumour risk, hypospadias repair usually in a staged approach, hormone replacement therapy for initiation and maintenance of puberty, and growth‐promoting strategies for managing short stature. The delivery of optimal care requires a close collaboration among paediatric endocrinologists, urologists, geneticists, gynaecologists, and psychologists [2].

### 5.1 Sex Assignment in MGD

The 45,X/46,XY genotypic mosaicism in MGD, in conjunction with variable prenatal androgen exposure to the developing gonads, results in varying degrees of atypia in the genital appearances, thereby causing significant clinical and ethical challenges during sex assignment. Medical and surgical considerations along with psychological aspects and ethical concerns need to be considered in decisions regarding sex assignment for patients with MGD through evaluations by the MDT. The considerations for sex assignment are summarised in Table 3. Assessment should consider likely prenatal androgenization of the brain, inferred from clinical virilization (e.g., phallic size or external masculinisation score) and supported by hormonal testing, recognising that these factors are contributory but not determinative of long‐term gender identity. Historically, a female gender assignment followed by prophylactic gonadectomy was the standard clinical approach, as this allowed appearance‐altering surgical procedures on the genitalia while mitigating the cancer risk associated with dysgenetic gonads. Early genital surgical procedures are controversial as they can cause permanent changes without children's understanding or consent [26, 27]. This is especially true for feminising surgeries due to their irreversible nature. The modern clinical practice focuses on patient‐centred care and advocates for the deferral of any irreversible interventions unless they are deemed clinically essential. It encourages open and honest discussions between healthcare providers, family members and the patient themselves when appropriate [28]. The decision‐making process should consider external genital anatomy, internal reproductive anatomy along with gonadal function, endogenous hormone production, fertility potential, sexual and urological function, malignancy risk, and cultural or familial values [5]. It is important to identify values including those of the patient wherever possible that might require psychological support. The evaluation of newborns with atypical genitalia needs extensive diagnostic tests that include genetic analysis, karyotyping, endocrine evaluation and imaging studies, to determine early management. Deferred sex assignment, whilst awaiting the results of evaluation with ongoing MDT discussions and family support is usually recommended [8, 29]. The decision to assign a male sex is usually considered, particularly for individuals with functional testes and predominantly male appearance of external genitalia. The presence of uterus together with nonfunctional gonads supports consideration towards female sex assignment [30]. The determination of sex in MGD cases requires careful balance between minimising the risk of gonadal malignancy, maintaining bodily autonomy and sexual function, all of which can contribute to good psychological adaptation and functioning. Best practices involve ongoing communication with the MDT and the family, alongside regular reassessment of psychosocial and medical indicators. Caregivers' perceptions of uncertainty about their child's DSD are highest soon after diagnosis, Thus, the initial diagnostic period is a critical time for psychological assessment and intervention, with parent illness uncertainty being an important clinical target [31].

**Table 3:** Considerations within the decision‐making process for sex assignment in MGD.

Considerations for sex assignment in MGD
External genital anatomy
Internal reproductive anatomy and gonadal function
Endogenous hormone production
Fertility potential
Sexual function
Urological function
Malignancy risk
Cultural and familial values
Prenatal androgen exposure*

* Assessment should consider likely prenatal androgenization of the brain, inferred from clinical virilization (e.g., phallus size/external masculinization score) and supported by hormonal testing, recognizing that these are contributory but not determinative factors in long‐term gender identity.

### 5.2 Gonadal Management and Tumour Risk

One of the foremost clinical concerns in individuals with MGD is the increased risk of gonadal tumours, most notably gonadoblastoma and germ cell neoplasia *in situ,* which are both premalignant neoplasms with the potential to become malignant, evolving into dysgerminoma [5, 32]. The presence of Y‐chromosomal material within a dysgenetic gonad, along with the presence of immature germ cells, is the primary risk factor for tumour development [5]. The tumour risk is influenced by factors such as age, location of the gonads, viability of the germ cells, and degree of gonadal differentiation. For individuals with 45,X/46,XY mosaicism, the lifetime risk of developing gonadal tumours is estimated at 15%–25%, with this risk rising with age, although the true incidence may be confounded by the historical practice of early gonadectomy and diagnostic challenges [11]. Although the risk is higher in older age, cases of malignancy have been described in young patients, but the incidence before the second decade of life is rare, and mortality associated with the early onset of tumours has not been reported [33]. The premalignant neoplasm risk was found to be highest (75%) in intra‐abdominal gonads, followed by inguinal (16%) and scrotal (9%) gonads, in a study describing patients with age at diagnosis between 1 month and 23 years [34]. Literature suggests that the risk is directly proportional to the degree of undifferentiated gonadal tissue present in the streak gonads and dysgenetic testes. Therefore, in those patients, with unlikely gonadal function, an early gonadectomy is usually considered. For those raised as female, bilateral gonadectomy is generally advised in early childhood. In those raised as males, streak or dysgenetic gonads are typically removed early due to the increased risk of cancer. However, if there is a normally descended, morphologically normal testis, preservation is usually advised to allow for endogenous testosterone production during puberty. This approach, however, necessitates strict lifelong surveillance, including self‐examination, and potential future biopsies due to the ongoing cancer risk [35].

For patients in whom gonads are retained, proactive surveillance for gonadal germ cell tumours can be challenging. Imaging modalities for intra‐abdominal gonads often lack sufficient sensitivity to reliably detect early malignant changes [36]. The alternative option is relocating the gonads into the labioscrotal folds to facilitate easier examination, imaging, and biopsy. However, biopsy may not reliably represent the entire gonad, and blood tumour markers are not elevated in most DSD‐ associated germ cell tumours. The decision to retain gonads should be made following comprehensive, multidisciplinary discussions with the patient and their family, weighing the benefits of endogenous hormone production against the potential risk of malignancy. Despite MGD being considered as a DSD with a higher risk for development of premalignant neoplasms there is no clear evidence to recommend removal or maintenance of gonads. Therefore, a shared decision‐making approach, inclusive of the risks and benefits of both options, should be used to make these decisions in an individualised manner.

### 5.3 Surgical Management in MGD

**Figure 1:**
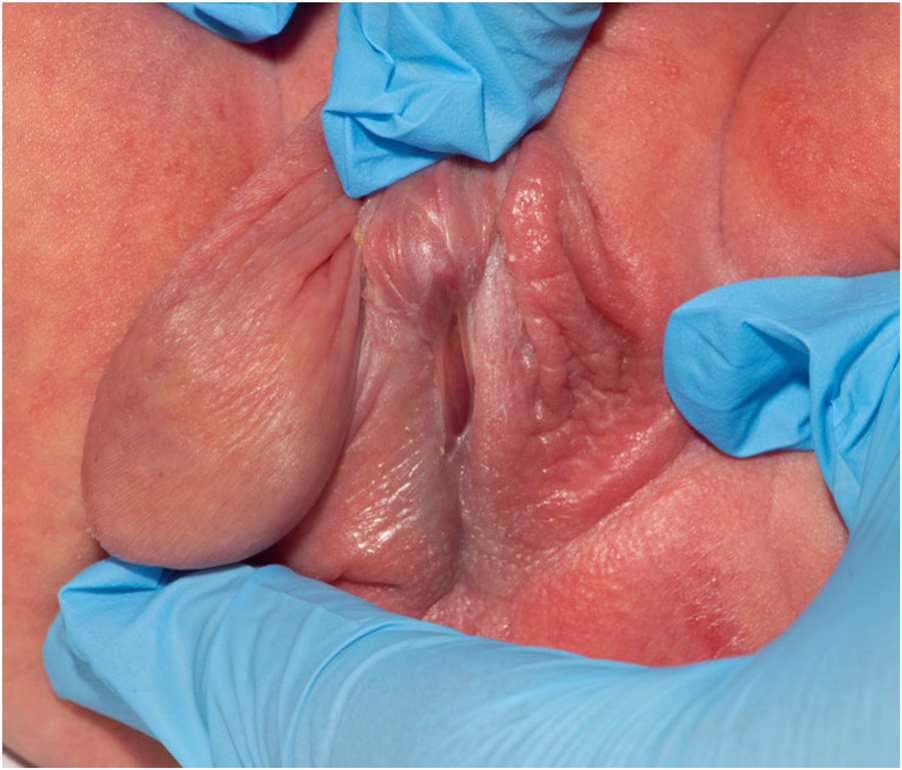
Typical external genital appearance of MGD patient with unilateral palpable gonad and perineal meatus with ventral penile curvature. These photographs were taken after full consent of the parents with permission for publication and stored appropriately in line with GMC regulations.

**Figure 2:**
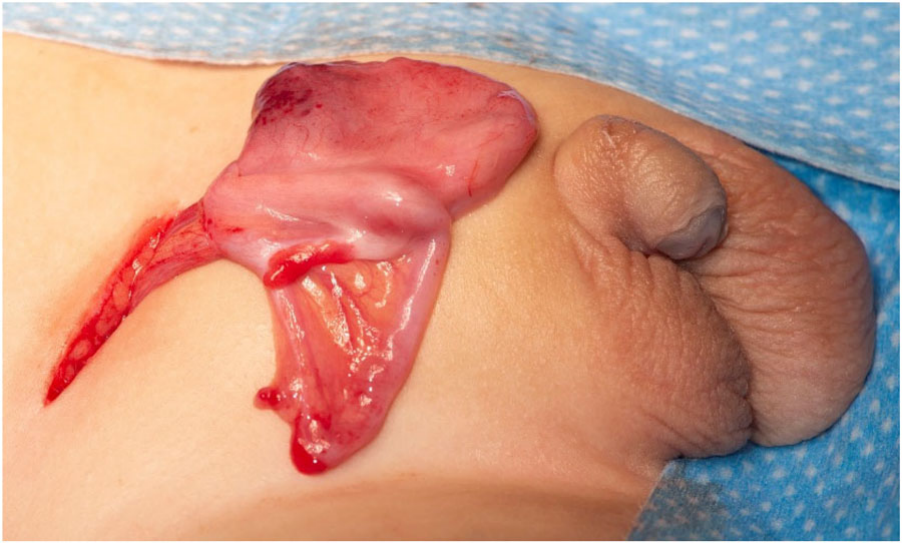
Abnormal gonad discovered at time of inguinal hernia repair as the initial presentation of MGD. These photographs were taken after full consent of the parents with permission for publication and stored appropriately in line with GMC regulations.

The role of the paediatric urologist in patients with MGD can be broadly divided into three aspects the first: as part of the wider MDT to agree the best sex of rearing based primarily on hormonal profile and karyotype. In the classic 45,X/46,XY babies with good testosterone production, this is likely to be male sex of rearing. The second role is clarification of the anatomy, which is usually coupled with excision of the intra‐abdominal streak gonad because of its malignancy risk. In the classic 45,X/46,XY patients, there will usually be a palpable testis in the hemiscrotum and an intra‐abdominal streak gonad on the contralateral side that is typically associated with a fimbria‐like structure and no normal vas deferens. Externally, there will be a perineal meatus, bifid scrotum, penoscrotal transposition, ventral penile curvature and an appearance similar to perineal hypospadias (Figure 1). Sometimes, the diagnosis of MGD is made during an inguinal herniotomy in a patient with proximal hypospadias when the gonad is seen intra‐operatively and recognised as a streak gonad rather than a normal testis (Figure 2). Internally, patients with MGD can have a prostatic utricle, which embryologically represents the caudal end of the Müllerian duct that regresses in boys under the effect of AMH, hence its association with MGD [37]. The third role of the paediatric urologist is related to the surgical intervention, which usually involves excision of the streak gonad initially, followed by managing the penoscrotal hypospadias in a staged approach similar to perineal hypospadias, only after the male sex of rearing has been agreed and confirmed with the family and the MDT. Occasionally, the prostatic utricle can become symptomatic, typically with recurrent epididymo‐orchitis and would need to be dealt with surgically especially if large [37].

The photographs (Figures 1 and 2) were included following thorough deliberation and in close collaboration with the parents or guardians. Given that the individual child is unable to provide informed consent, every effort was made to ensure that the images are non‐identifiable and ethically appropriate for publication.

### 5.4 Hormonal Therapies

#### 5.4.1 Growth Hormone Therapy

Short stature is a common clinical feature in individuals with MGD and is typically attributed to the 45,X cell line, and associated haploinsufficiency of the *SHOX* gene [38]. Growth trajectories often mirror those seen in Turner syndrome, with growth deceleration starting in early childhood. Recombinant human growth hormone (rhGH) therapy has been found to significantly improve growth velocity and final adult height in individuals with MGD, with outcomes similar to those observed in Turner syndrome [39]. As there are theoretical concerns that increased levels of GH and insulin‐like growth factor‐1 (IGF‐1) could potentially stimulate tumour development, careful, continued monitoring for any gonadal changes is important during treatment with rhGH [40]. Early intervention with rhGH, typically between the ages of 4 and 6 years is recommended to optimise linear growth before the epiphyseal closure [41].

#### 5.4.2 Puberty and Long‐Term Hormonal Replacement

Puberty and its associated surge in sex steroid hormones are imperative not only for the development of secondary sexual characteristics but also for their positive effect on body composition, bone mineral density cardiovascular, sexual and metabolic health [42]. Endogenous sex steroid hormone production in patients with MGD is typically limited to the synthesis of testosterone by the dysgenetic testis. Most individuals raised as males, with a retained dysgenetic testis, particularly those with an external masculinisation score (EMS) > 5/10 at birth, enter puberty spontaneously (60%–85%) [42, 43]. However, pubertal progression frequently stalls in most individuals suggested by rising FSH concentrations and declining testosterone concentrations [6, 43]. Moreover, inhibin B concentrations during early puberty may serve as a reliable predictor of testicular function and the need for testosterone replacement in the future [43]. The presence of Y‐chromosomal material increases the risk of germ cell tumours even in apparently normal testes located in the scrotal sac, and periodic surveillance by self‐examination is crucial [44].

Induction of puberty with either testosterone or oestrogen is essential for individuals with MGD who either have undergone gonadectomy or who do not experience spontaneous puberty. The primary aims of sex steroid treatment are the induction and maintenance of secondary sexual characteristics, preservation of bone mineral density, and support for psychosocial development.

Individuals with MGD assigned female at birth almost always require pubertal induction and hormone replacement therapy as prophylactic gonadectomy is performed in most, and spontaneous breast development is extremely rare [11]. In females, puberty is usually initiated with low‐dose transdermal or oral oestradiol around the age of 11–12 years, with gradual increases over 2–3 years to replicate normal pubertal development. With the onset of breakthrough bleeding, a progestogen is added to protect the endometrial lining if a uterus is present [45]. If the progestogen is given on a cyclical basis, a monthly withdrawal bleed will occur. It can also be given continuously if the patient wishes to remain amenorrhoeic. If a uterus is not present, patients can continue with oestrogen‐only HRT. Long term hormone replacement is recommended until the average age of natural menopause, approximately 50 years. Females can choose between standard HRT or combined contraceptives which can be administered as a pill or patch. A continuous combined oral contraceptive regimen, where the pill is taken daily without a pill‐free interval to provide uninterrupted oestrogen and progestogen exposure is recommended for optimal bone health. Options for HRT include tablets, patches, gels, sprays and intrauterine devices. This approach helps maintain stable hormone levels and optimises bone health in individuals without spontaneous ovarian function. Combined oral contraceptives containing either ethinylestradiol or 17β‐estradiol may be used, with the choice guided by patient preference, tolerance, and local prescribing practice.

For males with limited or absent testosterone production due to nonfunctional or absent gonads, testosterone replacement is initiated to induce puberty. This typically begins with low‐dose intramuscular injections administered every 4–6 weeks, with gradual dose escalation over several years to reach adult levels [46]. Transdermal testosterone may have a place in postpubertal maintenance regimes, following induction through puberty with intramuscular testosterone, and switching to an equivalent adult dose of the gel preparation [47]. Treatment with sex steroids is typically long‐term to maintain secondary sexual features, bone health, and overall health and vitality.

### 5.5 Psychosocial Support in MGD

Depending on the age of the patient at diagnosis, psychological approaches provided by the MDT and key professionals within the team will focus on parents, the patient, or both. Some studies have shown that parents of children with DSD experience high levels of distress and uncertainty in the neonatal and early childhood period, and about 50% have indicated benefit from psychosocial care, indicating that there is a gap in the provision of psychosocial care in DSD [31, 48, 49]. Support through a period of uncertainty aims to prevent excess fear and rigidity of thinking that assumes that “fixing” or “reversing” will be the outcome of healthcare. There should be a focus on the patient's present needs, including preparing for tests and investigations, caregiver relationships, and adapting to losses such as a sense of normalcy, including genital appearance, chromosomes, and expectations of fertility. Learning that other individuals and families have navigated life well with a similar diagnosis can be reassuring, therefore signposting to organisations such as the Turner Syndrome Society and DSD Families is important. Consultations with a specialist psychologist allow the parents or patient to explore their concerns, manage any maladaptive responses, and focus on ensuring that their actions align with their values. Different psychotherapeutic approaches may be utilised, depending on the needs of the parent(s)/patient. Throughout childhood and adolescence, proactive psychological care can be provided to prevent psychological adjustment difficulties, and to support meeting the key psychosocial developmental tasks and social milestones. Essentially, the parents may require supportive guidance to facilitate the child in growing up with an understanding of their body and understanding its possibilities and limitations [50]. Without this, parents might expect genital surgery to meet long‐term psychological needs [50].

### 5.6 Gender Identity in MGD

Gender incongruence is when there is a mismatch between an individual's gender identity and their sex assigned at birth. When gender incongruence leads to psychological distress or impaired functioning, this is called gender dysphoria [51]. The overall prevalence of gender incongruence among those with DSD is around 15% with rates among individuals with MGD reported at 12% or higher, as found in a recent systematic review and meta‐analysis [52]. The association of gender‐related distress in these patients can complicate psychosocial and psychosexual development in their early years. Due to the presence of a wide range of phenotypic variability in MGD patients, together with cultural, societal and stigma‐related factors associated with gender nonconformity, the actual prevalence might be underestimated. It is unclear whether dissatisfaction with sex assignment is more common in individuals with MGD assigned female or male at birth, although some clinical reports suggest that this might be more commonly observed when the sex assignment at birth was female [53, 54]. Nevertheless, it is critical for a coordinated MDT approach to discuss issues of potential gender identity in these patients, ensuring that the decisions on sex assignment and gender support are individualised, well‐informed, and respectful of the patient's evolving identity [2].

### 5.7 Fertility Considerations

The occurrence of spontaneous fertility in patients with MGD is extremely rare due to abnormal gonadal development. However, with assisted reproductive techniques such as testicular sperm extraction, rare instances of fertility have been reported [55]. The fertility potential of the gonadal tissue largely depends on the presence of germ cells. A study examining the histology of patients with DSD undergoing gonadectomy found that germ cells numbers were highest during earlier ages, and tend to decline with increasing age. In this study, among 44 patients with DSD due to various diagnoses, six patients had MGD, and all of them had germ cells at the median age of 15 months. This further complicates decision‐ making between early gonadectomy and retaining gonads [56]. Therefore, gonadal tissue cryopreservation during gonadectomy or gonadal biopsy, when the germ cell numbers are high, and the use of the tissue for future fertility when technological advancement potentially allows for in vitro maturation are being explored as options [57].

### 5.8 Long‐Term Follow‐Up and Transition

Long‐term follow‐up for individuals with MGD should include regular monitoring of growth, pubertal development, monitoring for bone mineral density, sensorineural hearing loss, and screening for associated health conditions linked to the 45,X cell line, such as cardiovascular or renal anomalies, and autoimmune conditions (notably autoimmune thyroiditis) [2]. Psychosocial support is equally crucial from the time of diagnosis and throughout life to help families and individuals address questions and challenges related to potential gender identity, self‐image, disclosure, and fertility [2].

For children and adolescents with MGD, transfer to adult care may be seen as a major change, as they generally have a long‐standing relationship with, and share a level of comfort, with the team looking after them since diagnosis [41]. Moreover, scarcity of adult specialists with in‐depth knowledge in DSD further hampers the transition process [6, 58]. Planning for transition should ideally begin as early as 12–13 years of age, and the European DSD consensus statement highlights early, staged hand‐over as the single most effective safeguard against loss to follow‐up [5]. A dedicated transition clinic or a joint paediatric‐adult endocrinology, urology and gynaecology model has been shown to improve treatment adherence [59, 60]. Tools covering information on diagnosis, self‐administration of hormones, contraception, fertility options, and mental‐health contacts have been published and validated for DSD and should be incorporated as a structured readiness checklist [61]. Bone mineral density, blood pressure, lipid profile, and glycated haemoglobin (HbA1c) should be recorded during the first adult‐clinic visit and monitored periodically thereafter. Self‐examination, testicular ultrasound, and/or tumour markers should be monitored in individuals in whom the gonads have been retained [42, 59]. Discussions on relationships, consent, and fertility preservation can be held during these transition clinics, along with the involvement of the wider MDT caring for the patient [56].

## 6 Long‐Term Outcomes

Short stature is a common feature among children with MGD, owing to haploinsufficiency of the *SHOX* gene [11]. Long‐term follow up studies involving large cohorts have shown progressive reduction in the height Z‐score with age. Treatment with rhGH is indicated in these cases, and an improvement in height SDS by 0.42 has been shown among treated patients [11, 43, 62]. The commencement of GH therapy during early childhood with optimal dosing can influence the final height [43, 62]. Testicular insufficiency develops in 60%–70% of individuals raised as males by the third decade, and infertility is the most commonly reported outcome. Rearrangements of the Y chromosome, particularly Yq loss, have been identified as a cause of the high incidence of infertility [43]. The risk of gonadal malignancy in patients with MGD has been reported to be around 15%–25% with the highest incidence in intra‐abdominal and dysgenetic gonads [11, 42, 63]. A recent population‐based study found a morbidity pattern in males with 45,X/46,XY karyotype, resembling classic Turner syndrome, with higher rates of hypertension, dyslipidaemia, and type 2 diabetes by age 40 [64]. Long‐term surveys have shown 80%–85% satisfaction rate with sex of rearing, but these individuals continue to have persistent concerns over body image and relationship prospects [65]. Short stature has been reported as a major cause of dissatisfaction among adults with MGD [43].

## 7 Future Directions

The future management of individuals with MGD is likely to move towards incorporating biomarker‐driven tumour surveillance and advanced fertility technologies [66]. Circulating microRNA (miRNA) tumour markers, such as miR‐371a‐3p have shown promise and can serve as liquid biopsies for early germcell tumour detection [67]. The approach towards gonadectomy has now shifted from prophylactic removal to personalised surveillance pathways [66]. Fertility preservation techniques have made significant strides, with testicular tissue cryopreservation for pre‐pubertal patients with 45,X/46,XY mosaicism now being offered in specialised centres, highlighting the importance of early fertility discussions [68]. A web‐based decision aid for shared decision making in DSD related gonadal and genital surgery has been published recently, and efforts should focus on further improving such tools [69]. Furthermore, data on long‐term patient‐reported outcomes in individuals with MGD, both with and without a history of genital surgery, are limited, highlighting the need for further research [70, 71].

## Conflicts of Interest

The authors declare no conflicts of interest.

## Data Availability

Data sharing is not applicable to this article as no databases were generated or analysed during the current study.
